# A Study of the Brain Abnormalities of Post-Stroke Depression in Frontal Lobe Lesion

**DOI:** 10.1038/s41598-017-13681-w

**Published:** 2017-10-16

**Authors:** Yu Shi, Yanyan Zeng, Lei Wu, Wei Liu, Ziping Liu, Shanshan Zhang, Jianming Yang, Wen Wu

**Affiliations:** 1 Department of Rehabilitation, Zhujiang Hospital, Southern Medical University, Guangzhou, 510282 China; 2 Department of Radiology, Zhujiang Hospital, Southern Medical University, Guangzhou, 510282 China

## Abstract

Post stroke depression (PSD) is a serious complication of stroke. Brain imaging is an important method of studying the mechanism of PSD. However, few studies have focused on the single lesion location. The aim of this study was to investigate the brain mechanism of frontal lobe PSD using combined voxel-based morphometry (VBM) and functional magnetic resonance imaging (fMRI). In total, 30 first-time ischemic frontal lobe stroke patients underwent T1 weighted MRI and resting-state fMRI scans. Clinical assessments included the 24-item Hamilton Rating Scale for Depression, the National Institutes of Health Stroke Scale, and the Mini-Mental State Examination. In our result, decreased gray matter (GM) volume in patients was observed in the prefrontal cortex, limbic system and motor cortex. The anterior cingulate cortex, selected as a seed to perform connectivity analyses, showed a greatly decreased functional connectivity with the prefrontal cortex, cingulate cortex, and motor cortex, but had an increased functional connectivity with the hippocampus gyrus, parahippocampa gyrus, insular, and amygdala. Stroke lesion location reduces excitability of brain areas in the ipsilateral brain. PSD affects mood through the brain network of the prefrontal-limbic circuit. Some brain networks, including motor cortex and the default mode network, show other characteristics of PSD brain network.

## Introduction

Post stroke depression (PSD) is a serious complication of stroke patients and occurs at a high incidence. Some studies have shown that at least 30–60% of post-stroke patients present symptoms of depression, which seriously restricts their rehabilitation^1,2^. At present, the PSD mechanism is unclear and treatment outcome is unsatisfactory, which greatly affects the prognosis of patients. PSD has become a prominent factor in stroke rehabilitation^3^. Therefore, understanding the mechanism of PSD is the key to precise targeted therapy^4^. Brain imaging technology provides an important means of studying brain network of PSD.

Many researchers have proposed different brain network mechanisms of PSD. Yang *et al*.^5^ suggested that PSD produces emotional disorders through a depression-related subnetwork, based on the emotional network^5^; In contrast, Vataja *et al*.^6^ suggested that the prefrontal subcortical circuits (such as caudate, pallidum and anterior capsule) in the left side are linked to PSD^6^. In our previous research, we also observed decreases in gray matter density (GMD) of the anterior cingulate cortex (ACC), dorsolateral prefrontal cortex (DLPFC) and hippocampus gyrus (HP) in PSD patients^7^. Other research groups have described the mechanism of an emotional circuit of PSD^8^.

However, most of the these studies (including ours) did not analyze the brain network of PSD in different patient types^9,10^. Instead, all types of patients were analyzed together, without paying attention to the basic factors of stroke such as type of stroke, location of lesion, size of lesion and duration of disease. Numerous studies have shown that some factors, including type of stroke and duration of disease, affect the development of PSD^11^, and that there is an association between PSD and some specific lesion locations or the hemisphere. Mood disorders of PSD are more likely to occur at specific lesion locations of stroke, such as the ganglia and left prefrontal cortex (PFC)^12^, which illustrates that different lesion locations have different effects on the disease. Therefore, putting all types of patients together for analysis results in a high heterogeneity of subjects. It is important to conduct brain network research on single types of PSD patients, which would improve the reliability of the conclusions.

In clinical practice, frontal lobe stroke is the most common type of stroke that causes PSD^13^. This may be due to the frontal lobe being closely related to the emotion, cognitive, memory and other advanced functions of the brain^14^. The frontal lobe is an important part of emotional processing and has a wide range of neural connections with many brain regions (e.g., thalamus, cingulate cortex and hippocampus). Consequently, frontal lobe damage is more likely to cause mood disorders^15^. We selected patients with frontal lobe strokes in this study as it is easier to explore the impact of lesion location on emotional networks, and then explore the brain network mechanism of PSD. In addition, the selection of a single lesion location allows a reduction in the heterogeneity of the study, and improves the reliability of the conclusion.

In brain imaging technology, voxel-based morphometry (VBM) can reflect the cortical volume density of the brain areas^16^, and resting-state fMRI (rs-fMRI) can reflect the functional connectivity (FC) of the brain network^17^, Both are useful methods to study the brain response of PSD. Seed-based analyses are frequently applied to resting-state data. The seed-based method is a hypothesis-driven approach wherein a seed region is selected as a reference, and the temporal correlations between the seed region and other brain regions are calculated. This allows the identification of a set of plausible FC alterations in neuropsychiatric disorders. Given that the ACC is commonly identified as the elementary structure related to emotional evaluation, it has been frequently chosen as the reference region in neuropsychiatric disorder studies. ACC, as part of the limbic system, is involved in the integration of the limbic cortex^18^. It is an important node of the emotional network including the default mode network (DMN) and the attention network, and has rich neural connections with the amygdala, thalamus and hippocampus. ACC also performs extensive information transmission with prefrontal cortex^19^. Therefore, the choice of ACC as a regions of interest (ROI) is an important entry way to explore the brain network mechanism of PSD.

To identify brain abnormalities of depression in a systemic manner, we employed the VBM and resting-state FC approaches to investigate structural alterations and potentially disrupted FC, respectively. To reduce heterogeneity, we chose patients with frontal lobe lesions of similar stroke type, duration, size of lesion and other basic factors. Our aim was to compare the brain response of PSD and non-PSD by grouping fMRI and T1 MRI datasets in frontal lobe lesions. We hypothesized that frontal lobe PSD is a unique feature of the brain network. Through this research, we hope to further the understanding of the mechanism of PSD, and provide a bridge for future research.

## Method and Subjects

### Participants

This study is part of the research project ‘Brain imaging of post-stroke depression’ (Clinical Research Foundation of Southern Medical University (CRFSMU) (LC2016PY037)). In this part of the project, we reviewed the charts of 133 patients admitted for ischemic stroke to the Zhujiang Hospital of Southern Medical University between Dec 2012 and Nov 2016. Briefly, all the patients and comparison subjects met the following criteria: meet the World Health Organization (WHO) criteria for the diagnosis of cerebral infarction based on both the presence of neurological symptoms and a compatible lesion, as demonstrated by magnetic resonance imaging (MRI); in the recovery period (3 months < disease duration < 1 year) with stable symptoms; have a single infarcted brain area (3–5 cm) in the right frontal lobe; National Institutes of Health Stroke Score (NIHSS) score < 6; be conscious and able to cooperate with the interview, provide informed consent, complete the scale evaluations and a clinical interview for the diagnosis of depression; Barthel Index score ≥ 60; aged 60 ~70 years old; without a history of hemorrhagic or ischemic stroke; without obvious cognitive dysfunction disorder and language understanding disorder; without a history of schizophrenia, major depression, anxiety, dementia, drug abuse or antidepressant use at stroke onset, or a family history of mental disorders; alcoholics or drug abusers removed; right-handed. After a detailed evaluation of inclusion and exclusion criteria, 30 patients were included in the study. The following information was collected from each subject: demographics (i.e., age, gender, education level and whether they lived alone) and stroke severity, as measured by the NIHSS at the time of admission to the hospital. Simultaneously, we obtained scores on the Mini Mental State Examination (MMSE) and the Barthel Index (BI).

All experiments and protocols were approved by the Ethics Committee of Zhujiang Hospital which is affiliated with the Southern Medical University, China^20^. According to the dictates of the State Council of China, each subject provided written informed consent after receiving detailed instructions and full explanations on the experimental procedures. All methods were performed in accordance with the relevant guidelines and regulations.

An experienced neuropsychologist performed the clinical interview to diagnose depression according to Diagnostic and Statistical Manual of Mental Disorders-IV (DSM-IV) criteria. The severity of depression was assessed using the 24-item Hamilton Rating Scale for Depression (HAMD-24). To be included in the depression group (PSD group) in our final analysis, participants had to meet DSM-IV criteria for depressive disorder and score at least 17 on the HAMD. The other participants were assigned to the control group (non-PSD group).

### Brain imaging

The experiment was performed in the Department of Radiology of Zhujiang Hospital, Southern Medical University, China. Anatomical scans of the brain were collected prior to stimulation imaging. Then, all subjects were subjected to a T1 weighted MRI and an rs-fMRI scan, each of which took 6 min.

Structural and functional scans were acquired with a 3.0 T Philips Achieva MRI System (Royal Philips Electronics, Eindhoven, The Netherlands) with an eight-channel head array coil equipped for echo planar imaging. The images were axial and parallel to the anterior commissure–posterior commissure line, which covered the whole brain. Structural images were collected prior to functional imaging using a T1-weighted fast spin echo sequence [repetition time (TR) = 25 ms, echo time (TE) = 3 ms, flip angle (FA) = 30°, field of view (FOV) = 230 mm × 230 mm, acquisition matrix = 192 × 256, slice thickness = 2 mm]. Blood oxygenation level-dependent functional imaging was acquired using a T2*-weighted, single-shot, gradient-recalled echo planar imaging sequence (TR = 2000 ms, TE = 40 ms, FA = 90°, FOV = 220 mm × 220 mm, acquisition matrix = 144 × 144, slice thickness = 1 mm). In addition, T1 MRI and fMRI image collection was preceded by five dummy scans to minimize gradient distortion.

### Voxel-based morphometry analysis

VBM analyses were carried out using Statistical Parametric Mapping software (SPM8: Wellcome Trust Centre for Neuroimaging, London, UK) run on Matlab 2010a (Math-Works, Natick, MA, USA). First, all images were checked for artifacts, structural abnormalities and pathologies. MR images were segmented into gray matter (GM), white matter (WM), and cerebrospinal fluid (CSF) using the standard unified segmentation module in SPM8. Second, study-specific GM templates were generated from the entire image dataset using the diffeomorphic anatomical registration through exponentiated lie algebra (DARTEL) method, an improved VBM method for greater accuracy in inter-subject brain registration. Third, after an initial affine registration of the GM DARTEL templates to the corresponding tissue probability maps in the Montreal Neurological Institute (MNI) space, non-linear warping of GM images were conducted to match the corresponding MNI space GM DARTEL templates. Fourth, images were modulated to ensure that relative volumes of GM were preserved following the spatial normalization procedure. Lastly, the modulated, normalized GM images (voxel size 1.5 × 1.5 × 1.5 mm^3^) were smoothed with an 8-mm full-width at half-maximum Gaussian kernel.

### Resting-state FC analysis

The fMRI image data were preprocessed and analyzed using the Data Processing Assistant for Resting-State fMRI (DPARSF, http://www.restfmri.net) by routines in MATLAB R2010a. The blood oxygen level-dependent (BOLD) time series preprocessing steps included removal of the first 10 volumes, slice-time correction, motion correction, intensity normalization, spatial smoothing, and linear high-pass temporal filtering. The first 10 volumes of each scan were discarded in order to eliminate any non-equilibrium effects of magnetization and to allow subjects to become familiar with the scanning environment. The motion time courses were used to select subjects’ head movements of < 2 mm in translation and 2° in rotation, which were used for further analysis (no subjects were excluded). Each individual’s functional images were normalized using the symmetric echo-planar imaging templates and resampled at a resolution of 3 mm × 3 mm × 3 mm. The normalized functional images were smoothed spatially using a 6 mm full width at half maximum (FWHM) Gaussian kernel. Finally, voxel-wise linear trend removal and temporal high-pass filtering (0.01 Hz < f < 0.08 Hz) were applied.

Data selection of both side of ACC for the ROI (3 × 3 × 3mm^3^) was based on the results of a previous MRI study^21^. MNI brain region coordinates were selected as the central voxel ROI (x = ±5, y = −10, z = 47). A function (FC) of DPARSF software was used. The individual time course of activity from the ROIs relative to the standard echo-planar imaging template for ACC was extracted, and six motion correction parameters and their global gray matter, white matter, and cerebrospinal fluid were removed. By analyzing Pearson correlation coefficients of the seed point and whole-brain voxel time series and using the Fisher’s Z-transformation of correlation coefficients into z values for standardization, brain functionality images for each subject were ultimately obtained.

### Statistical analysis

SPSS 18.0 software (SPSS, Chicago, IL, USA) was used to calculate descriptive statistics (mean ± SD) for psychophysical data. All statistical assessments were two-tailed, and we considered results to be significant at p < 0.05, consistent with the preliminary status of the trial.

VBM was used to compare gray matter volumes between the two groups using two-tailed, two simple t-test and corrected for multiple comparisons [false discovery rate (FDR), P < 0.05] in SPM 8. The FC value differences between PSD and non-PSD were calculated using two-tailed, two simple t-test, and corrected for multiple comparisons [FDR, P < 0.05].

## Results

### Demographic characteristics and clinical symptoms

We recruited 30 patients (female = 14) to the study. The mean age of the study sample was 65.23 ± 4.13 (range 61–69) years. Thirteen patients (43.3%) were diagnosed with PSD. A statistical difference was found in HAMD score between the PSD group and non-PSD group (p < 0.05). There were no significant differences in the basic data (i.e., age, sex, education, duration and whether they lived alone) and functional assessment scores (i.e., MMSE score, BI score and NIHSS score) between the PSD and non-PSD groups (p > 0.05) (Table 1).

**Table 1:** Summary of baseline characteristics of the 30 post-stroke patients.

Characteristic	Group, no. (%) or mean ± SD		p value
	PSD	Non-PSD	
n	13	17	—
Age, yr	64.39 ± 4.01	65.88 ± 4.23	0.17
Female. Sex	4 (30.8%)	10 (58.8%)	0.16
Education, yr	7.69 ± 3.44	9.24 ± 2.98	0.10
Duration, month	6.98 ± 3.80	7.14 ± 2.15	0.44
Live alone	3 (23.1%)	2 (11.8%)	0.23
Lesion size	3.98 ± 0.55	4.05 ± 0.63	0.38
HAMD score	20.18 ± 1.38	5.22 ± 1.92	<0.05*
MMSE score	22.20 ± 2.37	23.45 ± 1.94	0.06
BI score	72.76 ± 17.11	76.14 ± 20.16	0.31
NIHSS score	2.6 ± 1.2	1.8 ± 1.9	0.10

HAMD = Hamilton Rating Scale for Depression; BI = Barthel Index; MMSE = Mini Mental State Examination; NIHSS = National Institutes of Health Stroke Score; SD = standard deviation.

### Voxel-based morphometry analysis

**Figure 1:**
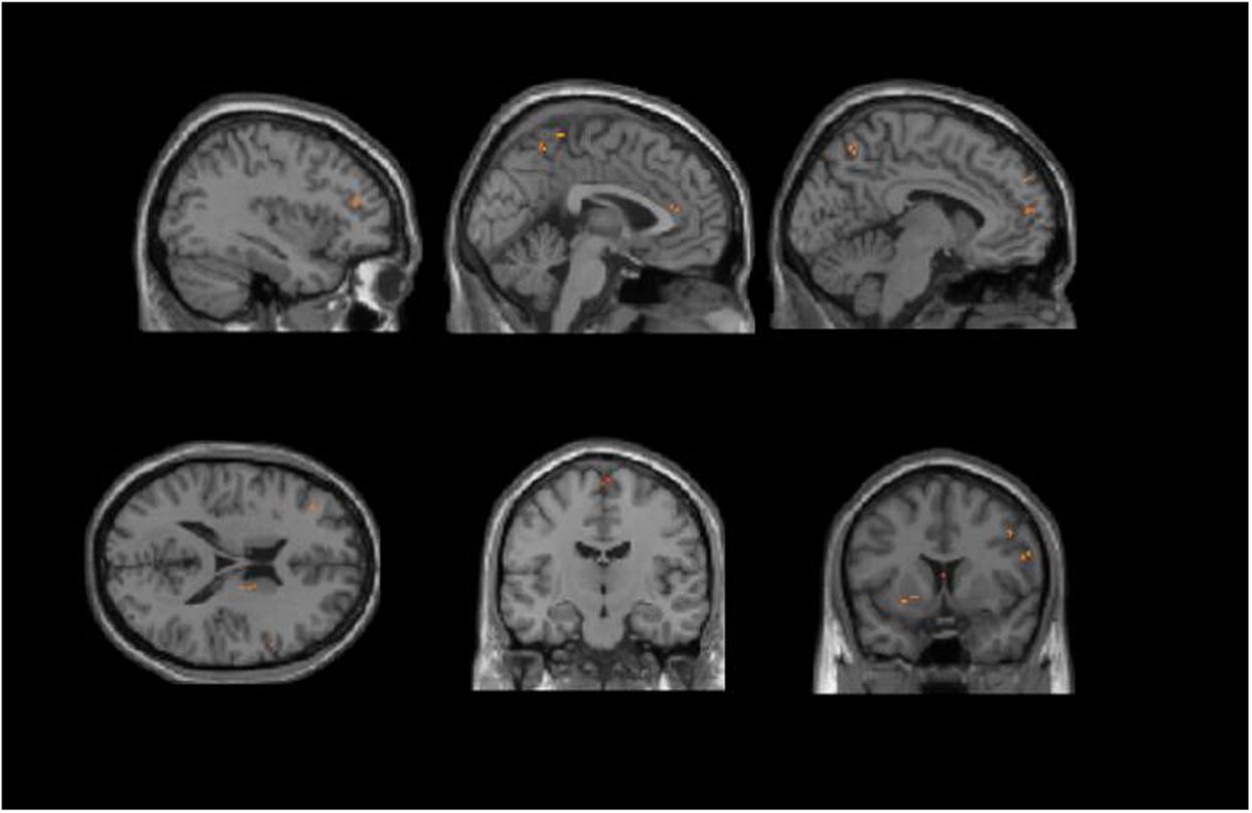
Regions showing significantly altered GM volume in PSD compared to non-PSD.

Compared with non-PSD patients, PSD patients displayed decreased volume in the prefrontal cortex [e.g., orbitofrontal cortex (OFC), dorsolateral prefrontal cortex (DLPFC) and ventromedial prefrontal cortex (VMPFC)], limbic system [hippocampus gyrus (HP), parahippocampa gyrus (PHP), ACC, mid-cingulate cortex (MCC), amygdala, mammillary body and insular], primary motor cortex (M1), primary sensory area (S1), secondary sensory area (S2), and supplementary motor area (SMA) (Fig. 1 and Table 2). Most of the decreased gray matter volume of the brain areas (especially in the limbic system) were located in the right hemisphere. The prefrontal cortex showed a decreased volume in both hemispheres.

**Table 2:** The locations of regions showing significantly altered GM volume in PSD compared to non-PSD. (P < 0.05, FDR < 0.05).

Brain region	BA	R/L	MNI			Voxel	Z-value
			X	Y	Z		
OFC		L	−9	49.5	−10.5	115	−10.9578
OFC	11	R	6	39	−30	216	−27.3044
DLPFC	10	L	−36	37.5	19.5	97	−9.2164
DLPFC	6	R	49.5	4.5	42	632	−22.681
VMPFC	9	L	−6	54	33	296	−11.0869
TP		R	40.5	−3	−15	86	−12.0504
HP		R	12	−10.5	−15	712	−13.8838
Amygdala		R	21	−6	−15	316	−18.8763
PHP		R	34.5	−24	−13.5	77	−39.1999
Mammillary Body		R	4.5	−10.5	−10.5	106	−15.443
Insular	13	R	30	21	−6	810	−16.1638
ACC	32	R	6	31.5	28.5	316	−20.6172
MCC	32	R	3	24	40.5	128	−10.7431
S2	6	R	57	−1.5	28.5	415	−21.7023
S1	2	R	42	−30	51	110	−11.0181
S1	3	L	−22.5	−31.5	66	95	−13.8333
M1	6	L	−18	−12	75	611	−19.0256
M1	44	R	54	4.5	22.5	118	−21.9849
Angular Gyrus		L	−34.5	−58.5	39	99	−15.2585
SMA	6	L	−3	−18	55.5	105	−11.0402
SMA	6	R	4.5	−15	63	513	−24.387
Superior Parietal Lobule	7	R	21	−69	60	289	−11.4009

Abbreviations: BA, brodmann areas; FDR, false discovery rate; MNI, Montreal Neurologi-cal Institute. OFC: orbitofrontal cortex, DLPFC: dorsolateral prefrontal cortex, VMPFC: ventromedial prefrontal cortex, M1: primary motor cortex, TP: temporal pole, HP: hippocampus gyrus, PHP: parahippocampa gyrus, ACC: anterior cingulate cortex, MCC: mid-cingulate cortex, PCC: posterior cingulate cortex, SMA: supplementary motor area, S1: primary sensory area, S2: secondary sensory area.

### Resting-state FC analysis

**Figure 2:**
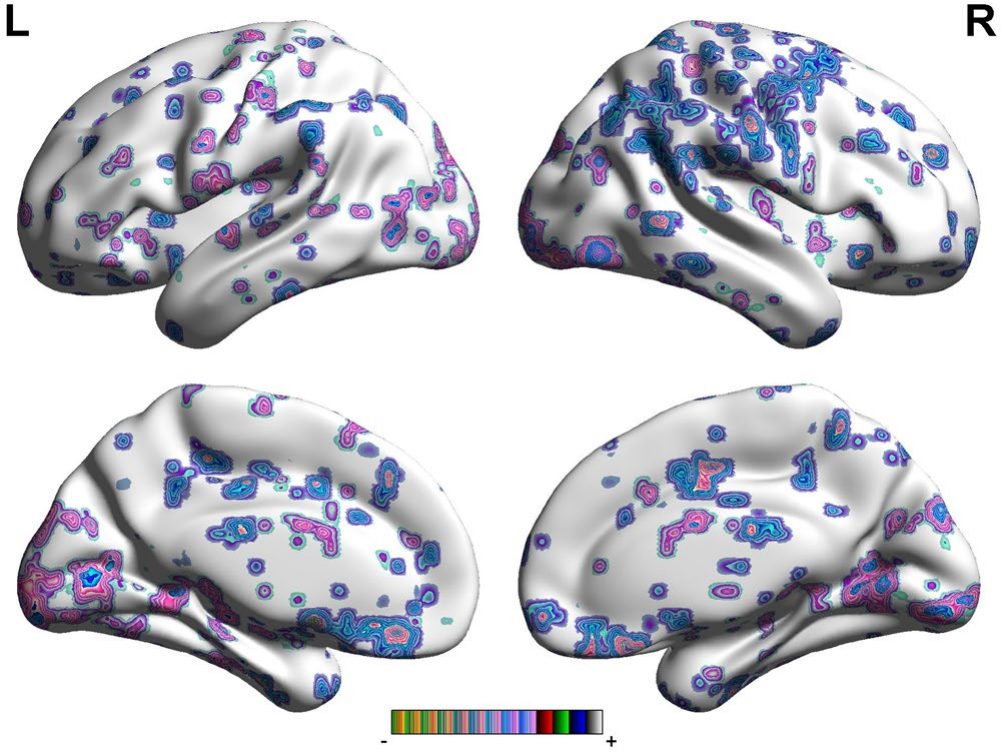
Regions showing significantly altered connectivity with the right ACC with PSD compared to non-PSD.

In follow-up analysis of resting-state brain activity, both sides of ACC were chosen as seed regions. FC maps for the whole brain were generated for each group. In the FC map of left ACC, Fig. 2 and Table 3 demonstrate that PSD displayed increased FC in the cerebellum, temporal pole (TP), PHP, insular, amygdala, HP and S2, when compared to the non-PSD. In contrast, PSD showed decreased FC in the OFC, DLPFC, VMPFC, MCC, posterior cingulate cortex (PCC), S1 and SMA. The most decreased FC of brain regions were located in the bilateral hemisphere; the most increased FC of brain regions were located in unilateral hemisphere.

**Table 3:** The locations of the regions showing significantly altered connectivity with the Left ACC in PSD compared to non-PSD. (P < 0.05, FDR < 0.05).

Brain region	BA	R/L	MNI			Voxel	P-Score
			X	Y	Z		
Cerebellum Posterior Lobe		R	36	54	−57	459	17.4951
Cerebellum Posterior Lobe		L	−42	−57	−51	347	9.1868
OFC	11	R	9	24	−18	821	−16.1373
DLPFC		L	−27	36	27	349	−16.3131
DLPFC		R	21	42	27	425	−6.8443
VMPFC		L	−12	48	9	330	−6.2226
VMPFC		R	9	42	−15	187	−8.3446
TP		L	−42	3	−27	233	6.3673
Brainstem		L	−6	−15	−24	93	6.0209
PHP	36	L	−36	−30	−24	510	14.5125
PHP	28	R	27	−24	−9	364	15.6093
MCC	32	R	15	15	36	235	−7.5942
MCC	9	L	−6	30	30	447	−13.6796
PCC	31	L	−12	−42	39	645	−7.5636
PCC	31	R	21	−36	39	369	−8.6697
Insular	13	R	33	21	9	240	12.3002
Amygdala		L	−18	−3	−12	265	8.8687
S2		R	60	−9	12	632	6.6426
S1	2	R	24	−39	72	457	−8.2304
SMA	24	R	6	−6	45	321	−5.9942
Angular Gyrus	7	R	33	−72	45	173	−8.0226

Abbreviations: BA, brodmann areas; FDR, false discovery rate; MNI, Montreal Neurologi-cal Institute. OFC: orbitofrontal cortex, DLPFC: dorsolateral prefrontal cortex, VMPFC: ventromedial prefrontal cortex, M1: primary motor cortex, TP: temporal pole, HP: hippocampus gyrus, PHP: parahippocampa gyrus, ACC: anterior cingulate cortex, MCC: mid-cingulate cortex, PCC: posterior cingulate cortex, SMA: supplementary motor area, S1: primary sensory area, S2: secondary sensory area.

**Figure 3:**
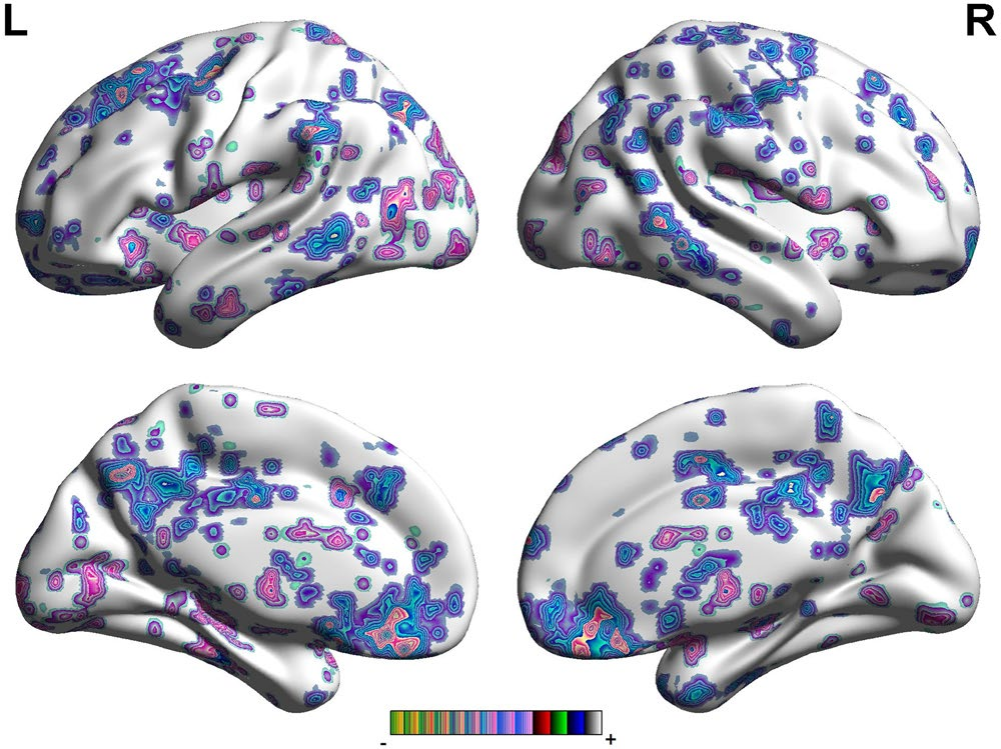
Regions showing significantly altered connectivity with the left ACC with PSD compared to non-PSD.

In the FC map of right ACC, Fig. 3 and Table 4 demonstrate that PSD displayed increased FC in the cerebellum, PHP, HP and insular, when compared to the non-PSD. Also, right ACC had a significantly decreased FC with prefrontal cortex, TP, MCC, PCC, thalamus, M1 and S1. In contrast to the FC map of the left ACC, the FC changes of the brain areas of the right ACC were located in the unilateral hemisphere.

**Table 4:** The locations of the regions showing significantly altered connectivity with the Right ACC in PSD compared to non-PSD. (P < 0.05, FDR < 0.05).

Brain region	BA	R/L	MNI			Voxel	P-Score
			X	Y	Z		
Cerebellum Posterior Lobe		R	39	−60	−45	510	17.8759
Cerebellum Posterior Lobe		L	0	−66	−42	269	18.347
OFC	11	L	−6	36	−15	137	−10.8707
VMPFC		L	−6	33	42	249	−7.9204
DLPFC		L	−36	6	45	387	−10.9616
TP	38	L	−27	15	−36	124	−11.2468
TP	38	R	45	24	−21	235	−15.5027
PHP	36	L	−36	−30	−21	453	6.2405
HP		L	−30	−15	−9	264	8.5213
MCC	24	R	3	0	42	817	−17.9107
PCC		R	−9	−33	48	93	−7.3258
Insular	13	R	33	27	12	311	19.6911
Insular		L	−27	−30	12	465	29.2977
Thalamus		R	9	−3	12	398	−11.0592
Brainstem		L	−9	−33	−6	564	7.0095
M1	4	R	57	−18	39	230	−9.0128
S1	2	R	51	−30	36	387	−8.8472
Angular gyrus	40	R	39	−57	42	168	−10.6127

Abbreviations: BA, brodmann areas; FDR, false discovery rate; MNI, Montreal Neurologi-cal Institute. OFC: orbitofrontal cortex, DLPFC: dorsolateral prefrontal cortex, VMPFC: ventromedial prefrontal cortex, M1: primary motor cortex, TP: temporal pole, HP: hippocampus gyrus, PHP: parahippocampa gyrus, ACC: anterior cingulate cortex, MCC: mid-cingulate cortex, PCC: posterior cingulate cortex, SMA: supplementary motor area, S1: primary sensory area, S2: secondary sensory area.

## Discussion

Stroke is prevalent worldwide, and has been ranked the third deadliest disease^22^. According to the WHO, 15 million people worldwide have a stroke each year^23^. However, the biological mechanism underlying PSD is still poorly understood. There is growing interest in probing structural and functional patterns of brain abnormities related to PSD. To our knowledge, the present study is the first to combine VBM and resting-state FC to investigate brain abnormalities with PSD in frontal lobe lesion. In PSD patients, we observed structural deficits in the right side of TP, HP, amygdala, PHP, mammillary body, insular, ACC, MCC, S2 and both sides of OFC, PFC, S1 and SMA. ACC is the important node of the emotional network and limbic system so was selected as a seed region for FC analyses. Regions with aberrant connectivity showed a large overlap with regions of decreased GM volume such as prefrontal cortex, the right side of the limbic system (e.g., MCC, insular, PHP and HP), and further corroborated previous findings that the prefrontal-limbic circuit is strongly implicated in PSD.

We observed no differences in the basic data (i.e., age, sex and educational level) between the PSD and non-PSD groups, which suggests that homogeneous data can reduce the heterogeneity between subjects and improve the reliability of the conclusion. Additionally, studies have shown that PSD is caused by major trauma and nerve damage caused by stroke, and concluded that stroke severity is the important factor for PSD^24^. However, in our study, there were no significant differences in NIHSS, MMSE and BI scores between the PSD and non-PSD groups. Despite both PSD and non-PSD patients suffering severe stroke trauma, there were some subjects who did not show depressive symptoms or had a low HAMD score. This suggests that nerve damage may not be a key factor for PSD development, and there must be other factors that influence its generation.

Our findings are in agreement with previous neuroimaging studies which showed that PSD displays significantly decreased GM volume in the ACC, MCC, PFC and insular. As an important node of emotional information exchange and integration, the decreased GM volume of the prefrontal cortex can seriously affect the integrity of the neural circuits, and also reduce the speed of emotional information transmission^25^. Furthermore, the brain regions with decreased GM volume were mainly distributed in the ipsilateral cerebral hemisphere, suggesting that the lesion location of the stroke had an inhibitory effect on the ipsilateral brain areas. Additionally, some motor and sensory cortices (e.g., M1, S1 and S2) had a decreased GM volume, which may explain the phenomenon of abnormal sensation and movement relaxation in PSD patients^26^.

Central to a broad array of cognitive, sensorimotor, affective and visceral functions, the ACC has emerged as a locus of information processing and regulation in the brain^27^. It is also involved in certain higher-level functions, such as reward anticipation, decision-making, impulse control and emotion^28^. The decreased GM volume in ACC would reduce the function of ACC acting on reward system, emotion, etc. In addition, in our study, ACC had significantly increased FC with HP, PHP, amygdala, insular and S2. As an important node of emotional transmission, abnormal FC of ACC may be linked to transmission of negative emotions.

### Prefrontal cortex

Our study found that both sides of the ACC had significantly decreased FC connection with OFC, PFC, MCC, PCC, S1 and angular gyrus. The lesion location would lose its original function due to stroke, and show a decreased FC with other brain areas. However, ACC also showed a decreased FC with PFC of unaffected hemispheres. This discovery has not been mentioned in previous studies. It is well known that the prefrontal lobe has a wide range of neural connections and complex structural schemas, as well as rich, complex bidirectional linkages involved in the processing of emotional information^29^. OFC is a combination of cortical areas that is involved in the integration of the prefrontal cortex, and is associated with higher levels of emotion. Decreased FC between OFC and ACC reduces the emotional information integration and transfer functions of OFC, and plays a role in the brain network of PSD. The left PFC and ACC are closely related to the reward system, which can promote the secretion of dopamine^30^. The decreased FC between PFC and ACC could reduce the occurrence of reward mechanism, and aggravate the negative state of the stroke patients. Impairments in the development of the ACC, together with impairments in the VMPFC, may constitute a neural substrate for socio-cognitive deficits in autism^31^, which would play a role in PSD and affect patient rehabilitation.

### Limbic system

The amygdala is considered part of the limbic system, and plays a primary role in the processing of memory, decision-making, and emotional reactions^32^. Studies show that damage to the amygdala can interfere with memory that is strengthened by emotion. Convergent evidence from therapeutics, neuroimaging and lesion studies suggests that amygdala disturbance is implicated in the pathophysiology of depressive illness^33^. Our finding of decreased GM volume in the right side of amygdala of PSD further supports the critical role of the amygdala in the network of PSD. ACC has neural connections with multiple brain regions such as HP, PHP and thalamus. The left side of the amygdala showed an increased FC with ACC, which may be related to increased negative emotions. Moreover, as the amygdala is closely linked to social phobia^34^, increased FC of amygdala would increase the level of social avoidance, thereby affecting the patient’s return to society and life.

The HP is a major component of the brains of humans, and is a critical part of the limbic system. The HP is thought to be related to recent memory and emotional reactions or control^35^. Increased FC between right ACC and HP would improve the speed of negative emotion transmission, and break down the abnormal emotion. Also, HP is involved in emotional memory, as well as the etiology and persistence of depressive symptoms. Our results suggest that the excessive activation of HP would increase the negative emotional memory, thereby making patients more anxious and pessimistic. Moreover, compared to the left HP, the right HP showed a decreased GM volume in PSD, which would affect the function of emotional reactions or control of the right HP, and play a role in brain network of PSD.

Thalamus is the most important part of the limbic system, and is associated with changes in emotional reactivity^36^. In emotional conduction, the thalamus has nerve connections with multiple brain areas. The medial dorsal nucleus makes connections with cortical zones of the prefrontal lobe^37^. The anterior nuclei connect with the mammillary bodies, and through them (via fornix), with the HP and the cingulate cortex (CC)^38^. Consequently, it is believed that the thalamus is the relay station of emotional conduction. In our study, ACC had a decreased FC with thalamus and mammillary body, which can affect or block the emotional pathway. Studies have also shown that the frontal lobe and thalamus together constitute the awareness system, which is the main center of spiritual activity^39^. Decreased GM volume in both brain regions may affect the awareness system, which may be the reason for the thinking-slow symptom of PSD.

The CC is another part of the limbic system. It receives the information from the thalamus and the neocortex, and projects to the entorhinal cortex via the cingulum. CC is involved with emotion formation and processing, and memory^40^. This role renders the cingulate cortex highly important in disorders such as depression and schizophrenia. The decreased FC between ACC and MCC would lead to a reduction in the function of emotion formation and processing. Several studies have suggested that the PCC plays an essential role in self-appraisal and internal monitoring, as well as emotional memory^41,42^. Compared with non-PSD, the decreased functional connection of PCC would reduce the emotion control and the speed of emotional memory processing.

The insula is located in the central part of the cerebral hemisphere, and is widely associated with other brain regions, such as sensory cortex, cingulate cortex and prefrontal cortex. The abnormal function of insular is a factor in the development of PSD. The insula posterior has been implicated in the detection and interpretation of internal bodily states, which is closely linked to anxiety and body sensitivity^43^. The present findings suggest that the structural deficits in the insula, and the increased FC related to the ACC, are centralized in the insula posterior region, and that these brain abnormalities may be associated with elevated sensitivity to anxiety. The insular is believed to be involved in consciousness and play a role in diverse functions usually linked to emotion. The anterior insular cortex is thought to be responsible for emotions and processes a person’s sense of disgust socially^44^. The increased FC between insular and ACC may aggravate disgust symptoms of PSD.

The temporal lobe is involved in emotional processes, and is responsible for recognizing familiar facial emotions and understanding a person’s emotions from their body posture. There is also some evidence that the TP may be involved in precipitating emotional empathy and enhancing mood stability^45^. We observed that right ACC had a decreased FC with both side of TP, suggesting that TP would reduce its function of emotion control and increase unstable negative nerve impulses. A significant phenomenon was found in the study, that is, the FC between TP and Left ACC and between TP and right ACC was opposite. PFC, ACC and TP belong to limbic system^46^. As an important node of the limbic system, PFC has an important role in the transfer of emotional information. Our findings suggest that frontal stroke lesions can interrupt conduction circuit of limbic system of the ipsilateral hemisphere. Because of that, the FC between TP and ACC of the ipsilateral hemisphere decreases, while the FC between TP and ACC of the contralateral hemisphere enhances compensatorily. At the same time, some scholars propose that the FC between ACC and TP is modulated by the frontal cortex^47^. Due to right frontal stroke, the FC between TP and ACC of the ipsilateral hemisphere is out of control, while that of the contralateral hemisphere appeared functional compensation. These results suggest that frontal lobe plays an important role in depression, which may be the reason of high incidence of PSD in frontal lobe.

HP and PHP are the relay station of emotional conduction, and directly connect with the temporal lobe. The damage from stroke evokes HP/PHP to transmit more negative emotions, leading to enhanced HP/PHP activation.

### Motor cortex and default mode network

Our results demonstrated that ACC had a decreased FC with M1 and SMA, suggesting that PSD may reduce the function of motor cortex. It may also explain why PSD patients have more severe motor impairment than the non-PSD patients^48^. PFC, PCC, angular gyrus and TP all belong to the default mode network (DMN)^49^. As DMN has the function of awakening and feeling emotion, decreased FC of DMN would reduce the level of arousal, whilst ignoring other people’s emotional changes. This may be why PSD patients are indifferent and avoid society.

## Limitation

Selection was based solely on lesion location, which reduced the heterogeneity of the subjects. The reliability of the conclusions would be improved by analyzing more lesion locations. Assessment was only performed on elderly patients, but younger patients may exhibit a different brain response. Moreover, the sample size of this study was small; future investigations need a larger sample size for statistically accurate analysis. In addition, multimodal brain imaging (i.e., diffusion tensor imaging) is a useful tool for studying the anatomical connectivity of the PSD brain, and will be included in future work.

## Conclusion

Stroke lesion location reduces the excitability of brain areas in the ipsilateral brain. PSD affects mood through the brain network of the prefrontal-limbic circuit. Some brain networks, included motor cortex and default mode network, show other characteristics of the brain network of PSD.
